# The pursuit of oncotargets through understanding defective cell regulation

**DOI:** 10.18632/oncotarget.189

**Published:** 2010-10-18

**Authors:** Meng Qiao, Qian Shi, Arthur B. Pardee

**Affiliations:** ^1^ University of California, Irvine Biological Chemistry, 140 Sprague Hall, 839 Health Sciences Rd, Irvine, CA 92697-1700; ^2^ Institutes of Biomedical Sciences, Fudan University,130 Dong An Road, Box 281, Shanghai, China 20003; ^3^ Dana-Farber Cancer Institute, 44 Binney St, Boston, MA. 02115

**Keywords:** oncotarget, cancer, biomarkers, therapy, systems biology

## Abstract

More effective anticancer agents are essential, as has too often been demonstrated by the paucity of therapeutics which preserve life. Their discovery is very difficult. Many approaches are being applied, from testing folk medicines to automated high throughput screening of large chemical libraries. Mutations in cancer cells create dysfunctional regulatory systems. This Perspective summarizes an approach to applying defective molecular control mechanisms as oncotargets on which drug discoveries against cancer can be based.

## INTRODUCTION

The molecular basis of cancer is becoming understood, and is thoroughly reviewed [1]. But the promise of cancer drug therapy is unfulfilled. ‘Magic bullets’ against the disease have not been discovered. Chemotherapy often only briefly extends life and has severe side effects. As examples, the combination of paclitaxel and anthracycline is often used against breast cancer, although it benefits only 30% of patients. Drugs are not effective against stage IV melanoma; about half the patients develop metastases [2].

Improvements of cancer treatment are sorely needed. A major problem is selective lethality against cancer cells. Many drugs kill cancer cells in culture and in mice, but also kill normal cells and have severe side effects. This limits drug dosage. Another problem is that cancers respond differently to chemotherapy, as do five principal subtypes of breast cancer [3]. Discovery and clinical testing of an effective anticancer drug is enormously difficult and expensive; developing a potential drug can cost over 800 millions of dollars. Millions of chemicals, randomly synthesized even without a lead compound, are being screened and tested with rapid endpoints such as their lethality to cultured cancer cells. It is timely to summarize attempts to develop cancer therapies from the abnormal molecular regulatory mechanisms that are necessary for progression of cancer.

## I. NORMAL CELL REGULATORY BIOLOGY AND ONCOTARGETS

Approaches to discovering oncotargets can be based upon biochemical pathways of small molecule, protein and nucleic acids synthesis and degradation. These are qualitatively similar between normal and cancer cells, but their regulatory processes, either lost or constitutive in cancers [4] are more likely to provide oncotargets.

Major molecular biological regulatory mechanisms were discovered fifty-plus years ago. These include covalent enzyme modifications catalyzed by kinases, non-covalent binding by small molecules that rapidly feedback inhibit their own synthesis, e.g., metabolic feedback inhibition, protein-protein interactions alone or in multiprotein complexes, control of gene activation by repression, and functional activities of membranes. Another level of regulation is transport of small molecules including metabolites, drugs and regulators through the lipid bilayer cell membrane. Lipophilic molecules diffuse into the cytoplasm, but charged metabolites such as amino acids and nucleotides can require specific and energy-requiring enzyme-like permease channels. As an example, a ring of core proteins with attached regulatory calcium ion binding proteins is a ‘gate’ for potassium ions [5]. These changed regulatory mechanisms can provide targets for discovery of new treatments.

## II. MOLECULES ESSENTIAL FOR CANCER CELL GROWTH AND/OR SURVIVAL

### Mutations

Cancer cells deviate from normal cells due to mutations. An advanced tumor is composed of cells that have undergone diverse and numerous mutations, creating cells with many different properties. Thousands of changed gene expression have been found, but most do not appear in most cancers and are not essential. They might be caused by secondary mutations. Mutations increase as cancer develops, at least in part because the DNA repair mechanisms are defective, creating genetic instability [6]. Telomere loss is also a mechanism for chromosome instability [7]. DNA structure is thereby changed and alters amounts and characteristics of enzymes and other proteins. These mutations can stimulate oncogenes or inactivate tumor suppressor genes. In extreme cases mutation completely eliminates production of a protein. Therapies are being designed that target repair mechanisms [8]). Targeting a cancer driven by an activating mutation of B-raf is showing promise. A highly potent and specific B-raf inhibitor induced regression of melanoma in 80% of patients bearing activating B-raf mutations [9].

Epigenetics, heritable changes other than of the genomic sequence, have recently become of major interest. Gene expressions can be modified by post-synthetic methylations of DNA or of methylation and acetylation of associated histones in chromatin of eukaryotes [10]. They are heterogeneous in patients [11]. Epigenetic modifications are involved in plasticity, the capacity of alteration of a cell's phenotype in response to changes in environment [12]. They can modify gene expressions for many cycles, as during differentiation of stem cells [13], and could become permanent, and thereby involved during cancer progression, in loss of cells ability to differentiate and become quiescent [14].

Epigenetic oncotargets are abundant [15] and are being applied for drug development [16]. Advancements that include targeting histone methylation [17], and mitochondria inner membrane transporters [18] may provide novel therapeutic strategies that compliment/synergize with the current available modalities.

Furthermore, cancer stem cells, a very small subpopulation of cells with different properties from the majority, appear to be fundamental to tumor progression. Cancers become resistant to treatment; the tumor can shrink but the patient will die because lethal stem cells survive. These are proposed to be crucial targets for therapy [19]. A strategy that includes finding oncotargets present in cancer stem cells may help alleviate this resistance to therapy. BB1608, a first-in-class cancer stem cell stemness inhibitor is in clinical trials with promising phase I data (Langebland, AACR, 2010). Development of methods for producing immortalized mammary stem cells that can self replicate and differentiate should greatly help discovery of stem cell oncotargets [20].

### Metastasis and oncotargets

Processes that can provide oncotargets are molecular changes that cause defective proliferation, block programmed cell death (apoptosis) under stress, production of new blood vessels (angiogenesis), and drug resistance, and escape of cells to initiate secondary tumors (metastasis).

Metastasis appears in cancer progression, and is a key step leading to malignancy and cancer lethality [21. Epithelial cells undergo transition (EMT) to a mesenchymal phenotype with loss of cell-cell adhesion, more motility and greater invasiveness. EMT involves several molecules that could provide oncotargets, such as the metalloproteases that decrease cell adhesion. As an example, kinase Akt1 was found to be hyperphosphorylated when breast cancer cells became metastatic [22]. This study illustrates the increased difficulty of treatment as a cancer develops: Akt is also a key regulator of cell growth, apoptosis and migration. It has roles in neurological and other diseases. There are three isoforms of Akt with some overlapping functions, although each isoform of Akt has unique downstream targets. Growth factors binding to receptor tyrosine kinases (e.g., IGFR, erbB2) recruit Akt to translocate to the cytoplasmic membrane where Thr308 on its activation loop and Ser473 on the hydrophobic motif are phosphorylated by PDK1 and mTORC2, respectively, leading to Akt activation.

Activation is reversed by phosphatases. PP2A dephosphorylates Thr308 of Akt. In a search for a phosphatase containing a PH domain that could co-localize with Akt near plasma membrane, PHLPP (PH domain leucine rich repeat protein phosphatase) was discovered to be the long sought after phosphatase for Ser473 of Akt [23]. PHLPP containing a PP2C domain is a member of the serine/threonine phosphatase family Expression of PHLPP protein and mRNA are much decreased in the metastatic cell line of the breast cancer progression series 21T [22]. There are two isoforms; PHLPP1*b* (SCOP) with an N-terminus extension is 1.5kb longer than PHLPP1*a*. Another PHLPP family member, PHLPPL that dephosphorylates Ser473 Akt was later identified [24].

The requirement of domains for PHLPP functioning is substrate dependent, as PH domain is essential for PKC and PDZ domain is required for Akt dephosphorylation. There are several highly conserved domains in PHLPP and PHLPPL, including PH domain, leucine rich repeat, PP2C domain and PDZ binding motif. PHLPP and PHLPPL specifically dephosphorylate distinct Akt isoforms. PHLPP exhibits high preference for Akt 2, 3, while PHLPPL has Akt 1, 3 as substrates. FKBP51 (FK506-binding protein 51) acts as a scaffolding protein for Akt and PHLPP and promotes dephosphorylation of Akt by PHLPP [25]. Besides Akt, PKC family members (PKC*a*, PKC*bII*) were also identified as substrates for PHLPPs [26].

PHLPP is a proteolytic target of b-TrCP (the substrate recognition subunit of SCF-Ub E3 ligase complex). Phosphorylation of four sites on the PP2C domain of PHLPP by casein kinase I and GSK3*b* will promote binding of PHLPP to b-TrCP that leads to ubiquitination and degradation of PHLPP [27].

There are over 400 known Ser/Thr kinases and far fewer (~ 30) Ser/Thr phosphatases. PHLPPs are predicted to have more substrates other than Akt and PKCa. Mst1 (Mammalian sterile 20-like kinase 1) was identified as a substrate for PHLPPs [28]. Mst1 participates in the regulation of mammalian cell morphology, motility and apoptosis by activation of mitogen-activated protein kinase (MAPK). Mst1 exists in auto-inhibitory dimers maintained by phosphorylation at Thr387. The PP2C domain of PHLPP interacts with Mst1 to dephosphorylate Thr387 in the inhibitory domain of Mst1 that leads to auto-phosphorylation on Thr183. FOXO, JNK and p38 pathways then become activated to promote apoptosis. Interestingly, Akt and Mst1 are reciprocally inhibitory. The Thr387 site of Mst1 can be phosphorylated by Akt and become inactivated. Mst1 could inhibit Ser473 Akt phosphorylation. Therefore, PHLPP, Akt and Mst1 form an intricate triangle to orchestrate the fine balance of cell survival and apoptosis in normal cells.

PHLPP, a tumor suppressor, is present in the majority of human tissues. Its expression is much decreased in human colon, pancreatic and gastric cancer patient specimens [28][29]. Over-expression of PHLPP reduces the tumorigenesis of glioblastoma and colon cancer cells in a xenograft nude mice model. In PHLPP1-null mice impaired capacity to stabilize circadian periodicity after light-induced resetting is observed [30].

In another PHLPP knockout mouse model Akt phosphorylation elicited by ischemia/reperfusion injury is enhanced; thereby Akt exerts its cardio-protective effect to reduce infarct size [31]. PHLPP (SCOP) was originally identified as an oscillating protein in the SCN nucleus that plays important roles in circadian rhythms. In addition, it regulates ERK signaling by interacting with Ras to modulate long-term memory [32]. How PHLPPs become activated is still under intensive investigation. As our understanding evolves, more PHLPP oncotargets will be identified and more function of PHLPPs will be discovered. The PHLPP-Akt-Mst1 pathway, deregulated in tumors, may result in more specific cancer therapeutics that minimize off target effects.

### Cell proliferation

Analysis of steps in the cell cycle, has been extensively investigated to identify oncotargets that lead to defective proliferation [33]. Extracellular factors greatly modify initiation of proliferation. Normal cells are arrested in G1 phase, prior to DNA synthesis, by contacts with adjacent cells and the extracellular protein matrix, but tumor cells continue to cycle. Epithelial cell adhesion molecule EpCAM is over expressed in cancer initiating cells. It is regulated negatively by the tumor necrosis factor alpha [34]. Lipophilic hormones (estrogen and androgen) permeate through cell membranes, bind to nuclear receptors, and activate gene expressions [35]. This process provides oncotargets for synthetic analogs that are applied against estrogen and androgen -receptor over-expressing tumors.

Extracellular binding of a growth factor protein to its trans-membrane receptor initiates cytoplasmic kinase cascades, such as MAP kinases that activate transcription factors in the nucleus. Epidermal growth factor (EGF) binds to the Her2/neu receptor on membrane of quiescent cells, which leads to the intracellular protein kinases activation. Her2/neu is in excess in about one third of breast cancers, and provides an oncotarget for drugs and for antibodies such as herceptin. The anticancer drug imatinib (Gleevec) that inhibits over active Bcr-Abl kinase in certain cancers provides an example of a widely applied therapy. Many kinases, including PI3K/Akt, have now become targets for therapy [36].

The by induction-repression mechanism of gene expression in the nucleus is vital for normal cell proliferation and function. The first step is transcriptional production of RNA, which is spliced into messenger RNA. Synthesis is countered by hydrolysis. mRNA provides information in their nucleotide sequence for translation catalyzed by ribosomes into the amino acid sequence of a protein, according to the genetic code.

siRNAs and microRNAs (miRNA) also regulate cell proliferation. These short double-stranded RNAs bind to mRNAs with complementary base sequence and target them for destruction or inhibit their synthesis. siRNA and miRNA have been widely used in cancer research to pinpoint the functions of many genes. This new area includes effects on epigenetics [37]. miRNAs play important roles in tumorigenesis and metastasis. siRNA based cancer therapeutics are being actively pursued [38][39]. A micro RNA, MiR-21 has been reported to be essential for proliferation of many cancers. MiR-21 is ‘addictive’; tumors regress unless it is produced [40]. Synthetic asymmetric RNAs show the promise to improve the current siRNA approach [41]. A possible complication is that Dicer and Drosha, key proteins involved in siRNA processing, were found to be down-regulated in cancers with poor outcome ([42]. These enzymes might become oncotargets of future therapeutics [43].

### DNA synthesis and mitosis as oncotargets

Numerous drugs that block DNA replication or cause damage are applied clinically [1]. The frequent uncontrolled DNA synthesis in cancer cells presents an oncotarget. Cyclin D and E are proteins synthesized in G1 phase of the cell cycle. They bind to and activate two cyclin dependent kinases, which phosphorylate retinoblastoma protein [44], thereby releasing DNA polymerase activity and entry into S phase. Cleaved low molecular weight cyclin E is found in many cancers, and is associated with poor prognosis [45].

Mitosis is a cycle related oncotarget. An early approach to drug discovery was to give a natural product such as one used in herbal medicine to a tumor-bearing animal and determine whether it is beneficial. One well-known example is Taxol (paclitaxel), whose oncotarget is microtubules in mitosis. Several chemically modified Taxol derivatives were synthesized, tested for effect on mitosis, and Taxol and Taxotere are presently applied as anticancer drugs.

b-Lapachone, in a folk medicine isolated from a tree's bark, selectively kills cancer cells in culture and in animals (46). It kills non-small lung cancer cells with elevated mitochondrial enzyme NQO1 by futile cycling of electron transport, which decreases production of ATP and NAD and produces toxic reactive oxygen [47]. b-Lapachone acts by at least two mechanisms because a four -fold higher dose also kills NQO1-negative cells [46]. Production of cellular energy (ATP) in normal cells is mainly by oxidation in mitochondria, versus increased glycolysis in cytoplasm of cancer cells. This is a classical example of a different biochemical process that provides an oncotarget [48]. Several drugs that act on mitochondrial functions are being developed [49].

### Protein synthesis and removal

Synthesis of enzymes and other proteins are central to regulation of biochemical processes. Enzymes that degrade proteins are closely regulated in normal cells. Excessive cancer cell proliferation depends upon short lived proteins. in particular cyclins, whose degradation can be defective. Protein synthesis is counterbalanced by degradation. Small proteases are not yet successful oncotargets; they can degrade both activating and inhibiting regulatory proteins, and drugs that inhibit them have complex effects. The proteasome, a large (about 50 subunit proteins) complex, mediates degradation of many proteins after they have been specifically tagged by attachment of ubiquitin proteins [50]. The proteasome inhibitor Velade (bortezomib) is applied clinically against multiple myeloma and mantle cell lymphoma; its combinations with other drugs are being tested against several types of malignancies. A protease blocks activation of NF-kB. Its inhibitors thereby are pro-apoptotic for cancer cells, and are being investigated.

### Cancer cell removal

Programmed cell death (apoptosis) counteracts uncontrolled proliferation of cancer cells. It can be lost in cancers, which provides an oncotarget: transcription factor NF-kB, linked to inflammation through TNF**a**, suppresses apoptosis of stressed cancer cells. The drug Go6976 blocks NF-kB activation, inhibits growth and causes degeneration of estrogen receptor negative breast cancers in vivo without detected damage to vital organs [51]. In vivo and in vitro experiments suggest that Go6796 blocks activation and anti-apoptotic activity of factor NF-kB by inhibiting protease caspase-8 [52]. Apoptosis of tumor cells was also sensitized by a polypeptide that specifically neutralizes anti-apoptotic protein MCL-1, and sensitizes cancer cells to drugs [53]. Drugs that promote respiration can selectively cause apoptosis of cancer cells [54]. Cells under stress release apoptotic protein cytochrome C from their mitochondria. siRNAs control apoptosis, among many processes [55]. Natural [56] and modified [57] siRNAs are being tested for specific anticancer activities

### Angiogenesis, another source of oncotargets

Blood vessel formation within a large cancer is required to provide oxygen and metabolites required to permit proliferation of internal cells. These vessels also allow released cancer cells to travel to distal sites and seed new cancers (metastasis), a process that correlates with lethality. Anti-angiogenesis agents such as Avastin can block tumor growth [58]. Pleiotrophin is a cytokine that induces tumor angiogenesis by binding to and inhibiting its transmembrane receptor tyrosine phosphatase. It also induces the association of b-catenin with cadherins EMT, and many other properties associated with tumor promotion [59].

## III. FUTURE ONCOTARGET RELATED BASIC RESEARCH AND THERAPEUTICS

Cancers progress from bad to worse. Biomarkers for early detection thus are highly important [60]. Biomarkers are also being sought for improved diagnosis, and for early determination of efficacy of therapy. Markers associated with melanoma include circulating tumor cells in blood [61], and molecular markers for tumor progression [62]. Apoptosis of cancer cells releases biomarkers into blood including postranslational modifications of proteins [63], which can be detected in very small non-invasive samples [64]. miRNAs in cancers are changed [65]. Urine of prostate cancer patients contains an siRNA biomarker; pooled samples were analyzed by differential display for selection of the most frequently changed biomarkers. Pooling should be valuable for finding frequently expressed potential oncotargets and to simplify research effort [66]. Biomarkers for colon and pancreatic cancers are found in fecal samples. Techniques for biomarker discovery include genome sequencing, arrays and differential display-related methods, proteins and phosphoproteins, antibodies, electrophoretic 2D gels, and nuclear magnetic resonance spectroscopy [67].

### Systems biology

The quantity and complexity of information about cancer presents a problem. Creation of cell phenotypes involves activities and regulation of groups of genes. More than one mutation is usually needed to make a hit, oncotargets are likely to act in sets, multi-drug combinations are superior to a single drug [68]. Comprehensive therapy based on oncotarget research should be bases on complex system models [69]. Systems biology is an approach to quantitate and visualize the massive information that links molecular biological pathways and their regulations. This modeling could provide a basis for studies of defective controls in cancers [70]. Negative and positive feedback loops [71] and bifan switching mechanism [72] have been discovered in cell signaling networks [73]. These can identify oncotargets as for metastasis [74] and for drug discovery [75].They are incorporated in stem cell systems [76].

Models of differentiation of normal cells from stem cells and regulations of their phenotypic changes [77] could be constructed based on systems biology. Pattern formation in oogenesis provides a dramatic example [78] Systems biology could move “reductionism” [79] into general biology [80]: ‘Nature’ is regulated by ‘Nurture’.

## References

[1] Weinberg RA (2007). The Biology of Cancer.

[2] Eggermont AM, Tesori A, Marsden J, Hershey P, Quirt I, Peterella T, Gogas H, MacKie RM, Hauschild A (2009). Utility of adjuvent systemic chemotherapy in melanoma. Ann Oncol.

[3] Rouzier R, Wagner P, Morandi P, Pusztai L (2005). Gene expression profiling of primary breast cancer. Curr Oncol Rep.

[4] Pardee AB, Stein GS, Pardee AB (2004). Misregulated fate - cancer. Cell Cycle and Growth Control: Biomolecular Regulation and Cancer.

[5] Weyand S, Iwatya S (2010). Old gate gets a new look. Science.

[6] Loeb L, Nishimura S (2010). Princess Takamatsu symposium on DNA repair and human cancers. Cancer Res.

[7] Murnane J (2010). Telomere loss as a mechanism for chromosome instability in human cancer. Cancer Res.

[8] Rowe BP, Glazer PM (2010). Emergence of rationally designed therapeutic strategies for breast cancer targeting DNA repair mechanisms. Breast Cancer Res.

[9] Smalley KSM, Flaherty KT (2009). Integrating BRAF/MEK inhibitors into combination therapy for melanoma. Br J Cancer.

[10] Goldberg AD, Allis CD, Bernstein E (2007). Epigenetics: a landscape takes shape. Cell.

[11] Heng HH, Bremer SW, Stevens JB, Ye KJ, Liu G, Ye CJ (2009). Genetic and epigenetic heterogeneity in cancer: a genomocentric perspective. J. Cell Physiol.

[12] Skipper M, Weiss U, Gray N (2010). Plasticity. Nature.

[13] Zaidi SK, Young DW, Montecino M, Lian JB, van Wijnen AJ, Stein JL, Stein GS (2010). Mitotic bookmarking of genes: a novel dimension to epigenetic control. Nature Reviews Genetics.

[14] Petronis A (2010). Epigenetics a unifying principle in the aetiology of complex traits and diseases. Nature.

[15] Mitsiades C.S., Anderson K.C. (2009). Epigenetic modulation in hematologic malignancies: challenges and progress. J Natl Compr Canc Netw.

[16] Balch C, Montgomery JS, Paik HI, Kim S, Kim S, Huang TH, Nephew KP (2005). New anti-cancer strategies: epigenetic therapies and biomarkers. Front Biosci.

[17] Shi Y, Lan F, Matson C, Mulligan P, Whetstine JR, Cole PA, Casero RA, Shi Y (2004). Histone demethylation mediated by the nuclear amine oxidase homolog LSD1. Cell.

[18] Xu X, Qiao M, Zhang Y, Jiang Y, Wei P, Yao J, Gu B, Wang Y, Lu J, Wang Z, Tang Z, Sun Y, Wu W, Shi Q (2010). Quantitative proteomics study of breast cancer cell lines isolated from a single patient: discovery of TIMM17A as a marker for breast cancer. Proteomics.

[19] Dick JE (2009). Looking ahead in cancer stem cell research. Nat Biotechnol.

[20] Zhao X, Malhotra GK, Lele SM, Lele MS, West WW, Eudy JD, Band H, Band V (2010). Telomerase-immortalized human mammary stem/progenitor cells with ability to self-renew and differentiate. Proc Natl Acad Sci U S A.

[21] Sleeman J, Steeg PS (2010). Cancer metastasis as a therapeutic target. Eur J Cancer.

[22] Qiao M, Iglehart JD, Pardee AB (2007). Metastatic potential of 21T human breast cancer cells depends on Akt/protein kinase B activation. Cancer Res.

[23] Gao T, Furnari F, Newton A. C. (2005). PHLPP: a phosphatase that directly dephosphorylates Akt, promotes apoptosis, and suppresses tumor growth. Mol Cell.

[24] Brognard J, Sierecki E, Gao T, Newton AC (2007). PHLPP and a second isoform, PHLPP2, differentially attenuate the amplitude of Akt signaling by regulating distinct Akt isoforms. Mol Cell.

[25] Pei H, Li L, Fridley BL, Jenkins GD, Kalari KR, Lingle W, Petersen G, Lou Z, Wang L (2009). FKBP51 affects cancer cell response to chemotherapy by negatively regulating Akt. Cancer Cell.

[26] Gao T, Brognard J, Newton AC (2008). The phosphatase PHLPP controls the cellular levels of protein kinase C. J Biol Chem.

[27] Li X, Liu J, Gao T (2009). beta-TrCP-mediated ubiquitination and degradation of PHLPP1 are negatively regulated by Akt. Mol Cell Biol.

[28] Qiao M, Wang Y, Xu X, Lu J, Dong Y, Tao W, Stein J, Stein GS, Iglehart JD, Shi Q, Pardee AB (2010). Mst1 Is an interacting protein that Mediates PHLPPs induced apoptosis. Molecular Cell.

[29] Liu J, Weiss HL, Rychahou P, Jackson LN, Evers BM, Gao T (2009). Loss of PHLPP expression in colon cancer: role in proliferation and tumorigenesis. Oncogene.

[30] Masubuchi S, Gao T, O'Neill A, Eckel-Mahan K, Newton AC, Sassone-Corsi P (2010). Protein phosphatase PHLPP1 controls the light-induced resetting of the circadian clock. Proc Natl Acad Sci U S A.

[31] Miyamoto S, Purcell NH, Smith JM, Gao T, Whittaker R, Huang K, Castillo R, Glembotski CC, Sussman MA, Newton AC, Heller Brown J (2010). PHLPP-1 Negatively Regulates Akt Activity and Survival in the Heart. Circ Res.

[32] Shimizu K, Mackenzie SM (2010). Storm DR SCO7P/PHLPP and its functional role in the brain. Mol Biosyst.

[33] Stein GS, Pardee AB (2004). Cell Cycle and Growth Control: Biomolecular Regulation and Cancer.

[34] Maetzel D, Denzel S, Mack B, Canis M, Went P, Benk M, Kieu C, Papior P, Baeuerle PA, Munz M, Gires O (2009). Nuclear signalling by tumour-associated antigen EpCAM. Nat Cell Biol.

[35] Thompson ME (2010). BRCA1 16 years later: nuclear import and export processes. FEBS J.

[36] Dillon RL, Muller WJ (2010). Distinct biological roles for akt family in mammary tumor progression. Cancer Res.

[37] Iorio MV, Piovan C, Croce CM (2009). Utility of adjuvant systems therapy in melanoma. Ann Oncol.

[38] Gondi CS, Rao JS (2009). Concepts in in vivo siRNA delivery for cancer therapy. J Cell Physiol.

[39] Ashihara E, Kawata E, Maekawa T (2010). Future prospect of RNA interference for cancer therapies. Current Drug Targets.

[40] Medina PP, Nolde M, Slack FJ (2010). OncomiR addiction in an in vivo model of microRNA-21-induced pre-B-cell lymphoma. Nature.

[41] Sun X, Rogoff HA, Li CJ (2008). Asymmetric RNA duplexes mediate RNA interference in mammalian cells. Nat Biotechnol.

[42] Merritt WM, Lin YG, Han LY, Kamat AA, Spannuth WA, Schmandt R, Urbauer D, Pennacchio LA, Cheng J-F, Nick AM, Deavers MT, Mourad-Zeidan A, Wang H, Mueller P, Lenburg ME, Gray JW, Mok S, Michael J., Birrer MJ, Lopez-Berestein G, Coleman RL, Bar-Eli M, Anil K., Sood AK (2008). Dicer, Drosha, and outcomes in patients with ovarian cancer. New England J Med.

[43] Shenouda SK, Alahari SK (2009). MicroRNA function in cancer: oncogene or a tumor suppressor?. Cancer Metastasis Reviews.

[44] Lundberg AS, Weinberg RA (1998). Functional inactivation of the retinoblastoma protein requires sequential modification by at least two distinct cyclin-CDK complexes. Mol Cell Biol.

[45] Wingate H, Puskas A, Duong M, Bui T, Richardson D, Liu Y, Tucker SL, Van Pelt C, Meijer L, Hunt K, Keyomarsi K (2009). Low molecular weight cyclin E is specific in breast cancer and is associated with mechanisms of tumor progression. Cell Cycle.

[46] Li CJ, Li C-Z, Pinto AV, Pardee AB (1999). Potent inhibition of tumor survival in vivo by b-lapachone plus taxol: Combining drugs imposes different artificial checkpoints. Proc Natl Acad Sci U S A.

[47] Blanco E, Bey EA, Khemtong C, Yang SG, Setti-Guthi J, Chen H, Kessinger CW, Carnevale KA, Bornmann WG, Boothman DA, Gao J (2010). Beta-lapachone micellar nanotherapeutics for non-small cell lung cancer therapy. Cancer Res.

[48] Vander Heiden MG, Cantley C, Thompson CB (2009). Understanding the Warburg effect: The metabolic requirements of cell proliferation. Science.

[49] Toogood PL (2008). Mitochondrial drugs. Currrent Opin Chem Biol.

[50] Lecker SH, Saric T, Goldberg AL, Hoffman R, Benz E, Shattil S, Furie B, Silberstein LE, McGlave P, Heslop H (2009). Protein degradation in cells. Hematology: Basic Principles and Practice.

[51] Singh S, Shi Q, Bailey ST, Palczewski MJ, Pardee AB, Iglehart JD, Biswas DK (2007). Nuclear factor-kappaB activation: a molecular therapeutic target for estrogen receptor-negative and epidermal growth factor receptor family receptor-positive human breast cancer. Mol Cancer Ther.

[52] Fulda S (2009). Caspase-8 in cancer biology and therapy. Cancer Lett.

[53] Stewart ML, Fire E, Walensky LD (2010). The MCL-1 BH3 helix is an exclusive MCL-1 inhibitor and apoptosis sensitizer. Nat Chem Biol.

[54] Bonnet S, Archer SL, Allalunis-Turner J, Haromy A, Beaulieu C, Thompson R, Lee CT, Lopaschuk GD, Puttagunta L, Bonnet S, Harry G, Hashimoto K, Porter CJ, Andrade MA, Thebaud B, Michelakis ED (2007). A mitochondria-K+ channel axis is suppressed in cancer and its normalization promotes apoptosis and inhibits cancer growth. Cancer Cell.

[55] Subramanian S, Steer CJ (2010). MicroRNAs as gatekeepers of apoptosis. J. Cell Physiol.

[56] Beezhold KJ, Castranova V, Chen F (2010). Microprocessor of microRNAs: regulation and potential for therapeutic intervention. Mol Cancer.

[57] Chang CI, Yoo JW, Hong SW, Lee SE, Kang HS, Sun X, Rogoff HA, Ban C, Kim S, Li CJ, Lee DK (2009). Asymmetric shorter-duplex siRNA structures trigger efficient gene silencing with reduced nonspecific effects. Mol.Ther.

[58] Naumov GN, Folkman J, Sraume O (2009). Tumor dormancy due to failure of angiogenesis: role of the microenvironment. Clin Exp Metastasis.

[59] Perez-Pinera P, Chang Y, Deuel TF (2007). Pleiotrophin, a multifunctional tumor promoter through induction of tumor angiogenesis, remodeling of the tumor microenvironment, and activation of stromal fibroblasts. Cell Cycle.

[60] Martin KJ, Fournier MV, Reddy GV, Pardee AB (2010). A need for basic research on fluid-based early detection biomarkers. Cancer Res.

[61] Keilholz U, Goldin-Lang P, Bechrakis NE, Max N, Letsch A, Schmittel A, Scheibenbogen C, Heufelder K, Eggermont A, Thiel E (2004). Quantitative detection of circulating tumor cells in cutaneous and ocular melanoma and quality assessment by real-time reverse transcriptase-polymerase chain reaction. Clin Cancer Res.

[62] Rother J, Jones D (2009). Molecular markers of tumor progression in melanoma. Current Genomics.

[63] Swaminathan R, Butt A, Gahan P (2006). Circulating Nucleic Acids in Plasma and Serum IV. Annals of the New York Academy of Sciences.

[64] Martin KJ, Graner E, Li Y, Price LM, Kritzman BM, Fournier MV, Rhei E, Pardee AB (2001). High-sensitivity array analysis of gene expression for the early detection of disseminated breast tumor cells in peripheral blood. Proc Natl Acad Sci USA.

[65] Calin GA, Croce CM (2006). MicroRNA signatures in human cancers. Nat Rev Cancer.

[66] Bai VU, Kaseb A, Tejwani S, Divine GW, Barrack ER, Menon M, Pardee AB, Reddy GP (2007). Identification of prostate cancer mRNA markers by averaged differential expression and their detection in biopsies, blood, and urine. Proc Natl Acad Sci USA.

[67] Han KQ, Huang G, Gao CF, Wang XL, Ma B, Sun LQ, Wei ZJ (2008). Identification of lung cancer patients by serum protein profiling using surface-enhanced laser desorption/ionization time-of-flight mass spectrometry. Am J Clin Oncol.

[68] Rouzier R, Wagner P, Morandi P, Pusztai L (2005). Gene expression profiling of primary breast cancer. Curr Oncol Rep.

[69] Hopkins AL (2008). Network pharmacology: the next paradigm in drug discovery. Nat Chem Biol.

[70] Ge H, Walhout AJM, Vidal M (2003). Integrating ‘omic’ information: a bridge between genomics and systems biology. Trends in Genetics.

[71] Brandman O, Meyr T (2008). Feedback loops shape cellular signals in space and time. Science.

[72] Lipshtat A, Jayaraman G, He JC, Iyengar R (2010). Design of versatile biochemical switches that respond to amplitude, duration, and spatial cues. Proc Natl Acad Sci U S A.

[73] Schrattenholz A, Soskic V (2008). What does systems biology mean for drug development?. Curr Med Chem.

[74] Erler JT, Linding R (2009). Network-based drugs and biomarkers. J Pathology.

[75] Berger SI, Iyengar R (2009). Network analysis in systems pharmacology. Bioinformatics.

[76] Macarthur BD, Ma'ayan A, Lemischka IR (2008). Toward stem cell systems biology: from molecules to networks and landscapes. Cold Spring Harbor Symp Quant Biol.

[77] Gerhart J, Kirschner M (1998). Cells, Embryos, and Evolution.

[78] Yakoby N, Bristow CA, Gong D, Schafer X, Lembong J, Zartman JJ, Halfon MS, Schupbach T, Shvartsman SV (2008). A combinatorial code for pattern formation in Drosophila oogenesis. Dev Cell.

[79] Woese CR, Goldenfeld N (2009). How the microbial world saved evolution from the scylla of molecular biology and the charybdis of the modern synthesis. Microbiol Mol Biol Rev.

[80] Bonner JT (2002). Lives of a Biologist.

